# Supplementary material for: The Walking Trail Making Test is more accurate than a Dual-Task Walking Test for screening the level of fall risk among community-dwelling older people
Source: PLoS One. 2026 May 21;21(5):e0348875. doi: 10.1371/journal.pone.0348875 (PMC13193416; doi:10.1371/journal.pone.0348875)
Supplement: S1 Results — (DOCX) [file pone.0348875.s001.docx]

**Supplementary results**

In the total sample, regarding TMT-A, 4 of the participants were classified as having a “deficit”: 1 (or 2.94%) in the low level of fall risk and 3 (or 5.56%) in the moderate level of fall risk (see Table 4). Regarding TMT-B, only 5 participants were classified as having a “deficit”: 4 (or 7.41%) in the moderate level of fall risk and 1 (or 7.69%) in the high level of fall risk (see Table 5). The overall distribution for each normative class did not show any statistical difference across the three levels of fall risk (for TMT-A, *X^2^*_(6)_ =3.58, *p*=.734 and for TMT-B, *X^2^*_(6)_ =6.55, *p*=.364).

**Table 4. TMT-A: repartition of each normative category corrected by age, gender and educational level, n (%).**

|  | Level of fall risk | | |
| --- | --- | --- | --- |
| Normative category  *Percentile, name* | **High**  (N=13) | **Moderate**  (N=54) | **Low**  (N=34) |
| <10, deficit | 0(0.00) | 3(5.56) | 1(2.94) |
| [10-25[, inferior | 0(0.00) | 3(5.56) | 1(2.94) |
| [25-75], average | 3(23.08) | 8(14.81) | 9(26.47) |
| >75, superior | 10(76.92) | 40(74.07) | 23(67.65) |

*Note.* In accordance with the normative standard proposed by Amieva et al. (2009), participants are considered in deficit from the 10^th^ percentile.

**Table 5. TMT-B: repartition of each normative category corrected by age, gender and educational level, n (%).**

|  | Level of fall risk | | |
| --- | --- | --- | --- |
| Normative category  *Percentile, name* | **High**  **(N = 13)** | **Moderate**  **(N = 54)** | **Low**  **(N = 34)** |
| <10, deficit | 1(7.69) | 4(7.41) | 0(0.00) |
| [10-25[, inferior | 0(0.00) | 3(5.56) | 3(8.82) |
| [25-75], average | 7(53.84) | 17(31.48) | 10(29.41) |
| >75, superior | 5(38.46) | 30(55.56) | 21(61.76) |

*Note.* In accordance with the normative standard proposed by Amieva et al. (2009), participants are considered in deficit from the 10^th^ percentile.
