# Supplementary material for: The Walking Trail Making Test is more accurate than a Dual-Task Walking Test for screening the level of fall risk among community-dwelling older people
Source: PLoS One. 2026 May 21;21(5):e0348875. doi: 10.1371/journal.pone.0348875 (PMC13193416; doi:10.1371/journal.pone.0348875)
Supplement: Attachment — Submitted filename: 07 - Responses_2_Reviewers.pdf [file pone.0348875.s002.pdf]

Responses to reviewers

We would like to express our gratitude to the reviewers, who provided insightful and constructive comments relative to our initial submission. Based on the reviewers' comments, we realized the topic addressed was too broad, and the statistical analysis sometimes inadequate to the problematic. Consequently, we completely reconsidered the reach and meaning of our initial results. The re-submitted manuscript is a **new manuscript**, with different purpose, objectives and analyses. It is, however, based on the same dataset, the same cognitive-motor tasks, and with similar problematic about fall-related outcomes. The current manuscript now focuses on the detection performance of the Walking-Trail Making Test (W-TMT), an *incorporated* cognitive-motor task, to classify different levels of fall risk and detect people at high level of fall risk, relative to the 6 Meters Walking Test (6MWT) in its Dual Task (DT) condition, an *additional* cognitive-motor task. After close look to the literature, a research question about the level of fall risk proved to be much more innovative than a question about the history of falling. The present manuscript was rewritten considering the reviewers' comments and suggestions.

Since we went for a completely new manuscript, we chose not to provide a point-by-point response to the reviewers, because some points became irrelevant in the new manuscript. Instead, we chose to synthetize our response by main topics, to illustrate to the reviewers how their comments were considered to build this new manuscript. The following table summarizes responses to both reviewers sorted by topic. Section and lines in which a modification was implemented are indicated directly after the response in italic with blue ink.

1. MAJOR comments

**Topic #1**<br>Objectives & Application in the community – Novelty of the study – Justification of tasks comparison		
**Reviewers' comments**		**Response**
Reviewer #1	"The manuscript contains excessive nonessential information, making the study's main focus unclear. Please summarize each paragraph within approximately 10 lines, emphasizing key points."<br>"The intended application of W-TMT-B in the community is unclear. 1) If the aim is to use W-TMT-B as a stand-alone fall screening tool, its discriminative ability should be compared with that of existing clinical screening tools. 2) If the aim is to add W-TMT-B to existing tools, the improvement in detection performance after adding it should be evaluated. In either case, analyses should be selected in alignment with the intended clinical application."<br>"The rationale for comparing W-TMT-B with the 6MWT is not clearly explained from a clinical perspective."	Several modifications were implemented in the new version of the manuscript to clarify the research question and the main objective of the study. These modifications included the suppression of non-essential information (e.g. definition of well-known concepts; notion of active/inactive older adults, etc.). This suppression led to reduce the manuscript section's length. More precisely, the total length of the introduction section went from 88 to 71 lines, and the discussion section went from 144 to 127 lines.<br>To **clarify the main topic** of the new manuscript, a focus was made on the level fall risk only. Indeed, in such retrospective design, focusing on the level of fall risk given by a multi-factorial and validated screening tool enhances the relevance of the study compared to history of fall alone. A "gold standard" tool recommended by the French National Health Insurance Fund was used to determine three levels of fall risk (low, moderate, high): the Health Examination Center Fall Risk

Reviewer #2	"In any case, it is essential for the authors to explicitly indicate the remaining research gaps, clearly define the research objectives based on the research gaps, and also choose the statistical methods to address the objectives."<br><br>"The authors describe previous studies using W-TMT as follows: 'Findings indicate that going from a simple condition containing only numbers to a more complex condition containing both numbers and letters enhances the ability to discriminate fallers, especially in active elderly people [38,39]'. This indicates that, even though the number of studies is limited and there is a difference in gait path between the previous studies and the present study, the research questions of this study (1 and 2) have already been clarified essentially."	screening tool (Bongue et al., 2011). This screening tool is described more precisely in this new version of the manuscript. *Materials & Methods section, lines 179-192*<br><br>**GAP #1**: As noted by Reviewer #2, previous works using the same type of cognitive-motor task and population already assessed history of fall retrospectively (Schott, 2015) and fall rate prospectively (Yamada & Ichihashi, 2010). A novelty of the present study is the use of the linear version developed by Alexander et al., (2005), named "Walking Trail Making Test", compared to the 5x5m square version of the task named "Trail Walking Test", for which no study has focused on fall-related outcomes.<br><br>This version of the task was chosen in regard of the **clinical intended use**, which was also highlighted in the new version of the manuscript. Indeed, in the community, the main clinical application intended for the W-TMT could be the screening of the level of fall risk at the beginning of a physical activity program for fall prevention. For older adults with a low or moderate level of fall risk, a physical activity program may be sufficient to reduce the rate of falls. For those at high level of fall risk, they may be referred to physicians for further assessment and the implementation of individualized multifactorial interventions to complement the physical activity program. *Implemented in the Discussion section, lines 396-403 and 494-498.*<br><br>Consequently, the rapidity of installation and administration is a key criterion for using this assessment in future baseline evaluation sessions with 10-15 older people by session. The version of the task used in the present study has the advantage of being simpler and quicker to set up and requires less time to complete than the Trail Walking Test version. In fact, the mean execution times for both versions of the task observed are:<br><br>Trail Walking Test, part B:<br>- Yamada & Ichihashi (2010): 66±37.6s for non-fallers and 91±33.7s for fallers.<br>- Schott (2015): 35.9±22.3s for non-fallers and 48.9±27.9s for fallers.<br><br>In the present study using W-TMT-B:<br>- From 20.16±7.90s for people at low level of fall risk to 30.69±15.45s for people at high level of fall risk. *Results section, lines 334-337*<br><br>**GAP #2:** To this date, no study compared two types of cognitive-motor tasks in their ability to detect the level of fall risk. Additional cognitive-motor tasks such as the 6MWT-DT are limited in terms of replicability, because they are exposed to prioritization strategies (i.e. participants can focus more on the cognitive or on the motor task, neglecting the other). Incorporated cognitive-motor tasks are an interesting alternative to additional DT, because the cognitive and motor tasks are interdependent. The single and shared overarching goal excludes any possibility of prioritization strategy (Herold et al., 2018). *Implemented in the Introduction section, lines 96-98*<br><br>In conclusion, the objectives of the study were clarified relative to theses research gaps, focusing on the two most complex conditions of the two cognitive-motor tasks (W-TMT-B and 6MWT-DT) as follows: to evaluate and compare the detection performance of two different cognitive-motor tasks in the screening of a high level of fall risk in community-dwelling older people. The W-TMT is used as an incorporated cognitive-motor task, and a simple walking DT, is used as an additional cognitive-motor task. *Implemented in the Introduction section, lines 122-125*

<table>
<tr><th colspan="3">Topic #2
Statistical analysis inadequate to the research question and suggestions for reconsidering the conclusions</th></tr>
<tr><th colspan="2">Reviewers' comments</th><th>Response</th></tr>
<tr><td>Reviewer #1</td><td>"The study essentially shows that W-TMT-B execution time differs between fallers and non-fallers. The authors should calculate the AUC for fall status as the outcome. Furthermore, if they intend to claim that W-TMT-B is superior to the 6MWT for detecting falls, the AUCs of both tests should be compared."

"As noted in the Major Comments, the current statistical approach is inadequate. Without at least reporting the AUC for fall status, readers cannot assess the clinical value of W-TMT."

"Hypothesis iii corresponds to an equivalence test. In a superiority test, a non-significant result does not imply no difference. Appropriate statistical methods for testing this hypothesis were not used."</td><td rowspan="2">The ROC analysis suggested by reviewer 1 was performed to evaluate the detection performance in the screening of people at high level of fall risk for the most complex condition of both tasks (W-TMT-B and 6MWT-DT). A new paragraph describing this analysis was added in the Materials & Methods section, lines 356-363

Results of this new analysis showed that the W-TMT-B detected people at high level of fall risk significantly better than chance (AUC=.46, 95%CI=.30-.62, p=.61). In contrast, for the 6MWT-DT task, this detection ability was not significantly better than chance (AUC=.72, 95%CI=.53-.90, p=.02).

To compare the detection performance of the two cognitive-motor tasks, a Delong test was performed. Results showed a significant difference between the two tasks (AUC difference=.26; 95%CI=.03-.49; Zscore=2.18, p=.03) demonstrating the relevance of using the W-TMT-B over the 6MWT-DT for classifying the three levels of fall risk. Results section, lines 356-363

Regarding the ordinal nature of fall risk levels, a more suitable statistical analysis was implemented. A c-index analysis was performed to evaluate the ability to classify the three levels of fall risk for both tasks most complex conditions (W-TMT-B and 6MWT-DT) (Van Calster et al., 2012). A new paragraph describing this analysis was added in the Materials & Methods section, lines 356-363

Results of this analysis showed that the W-TMT-B was able to classify individuals relative to their level of fall risk significantly better than chance in 63% of cases (c-index=.63; 95%CI=.52-.74; p=.02). In contrast, for the 6MWT-DT task, this classification ability was not significantly better than chance (c-index=.48; IC%95=.38-.59; p=.73).

To compare the classification performance of the two cognitive-motor tasks a pairwise comparison based on a Bootstrap test was performed. Results showed a significant difference between the two tasks (c-index difference=.15; 95%CI=.01-.29; p=.03), demonstrating the relevance of using the W-TMT-B over the 6MWT-DT for classifying the three levels of fall risk. Results section, lines 347-353</td></tr>
<tr><td>Reviewer #2</td><td>"Moreover, regarding purpose 3, it is necessary to select an appropriate statistical method to demonstrate that W-TMT is more suitable for evaluating fall risk than the dual-task condition of 6MWT."

"More specifically, although this study indicates a significant difference in W-TMT between fallers and non-fallers, it was an overstatement to claim that W-TMT can discriminate between these groups. Although the findings might show potential utility for risk identification, they support only the presence of an association (or a difference). Additional analyses would be required to address the research question more directly. In addition, since the analyses of W-TMT and 6MWT were conducted independently, a result such as one was significant and the other was not or may not be sufficient to demonstrate that W-TMT is superior to the dual task. Therefore, the results do not support the conclusion at present."

"The reviewer suggests performing a logistic regression analysis. For instance, this could involve comparing models with the presence or absence of falls as the dependent variable, including: a univariate model with W-TMT performance as the independent variable, a univariate model with 6MWT as the independent variable, and a multivariable model incorporating W-TMT, 6MWT, and covariates. It depends on the result, and this is just an example, but this approach could help demonstrate that 'W-TMT can distinguish between falls and non-falls' and that 'W-TMT is more useful as a fall assessment tool than 6MWT (DT condition)'."</td></tr>
</table>

2. MINOR comments

Topic	Reviewer #1	Reviewer #2	Response
Covariates for ANCOVA	"Only walking speed was used as a covariate. If causal inference is intended, known confounders (e.g., age, medication use, depression, comorbidities) should be adjusted for."		Based on previous studies on the W-TMT, only walking speed was introduced as a covariate, as it is a relevant indicator of functional status and contributes to the performance in the task (Perrochon & Kemoun, 2014; Persad et al., 2008).
Exclusion criteria	"The reason for excluding older adults at high risk of falls is unclear. The potential bias introduced by this exclusion should be discussed."		This ancillary study is based on the 13EVAL study inclusion/exclusion criteria. The objective of the 13EVAL study is to evaluate the effects of two primary fall prevention programs based on physical activity. This point has been clarified in the *Materials & Methods section, lines 132-137*<br>People who had three or more falls in the previous year were excluded to ensure that the physical activity proposed in the programs is safe and adapted.<br>It is important to note that the high fall risk category mainly includes people who have fallen only once (61.54%). *Results section, Table 2, lines 319-323*<br>Thus, the potential bias induced by the exclusion of people who had three or more falls in the previous year is reduced.
Other modifications	"The introductory sentences before stating the study's objective are unnecessary. Please concisely describe the knowledge gap related to W-TMT."<br>"There are two outcomes (fall history and classification based on fall risk). In the abstract, present only the primary outcome and its result."<br>"The meaning of "active" is unclear from the context."<br>"The phrase "both ANCOVAs" is ambiguous."		All these modifications have been implemented in the new manuscript. *Abstract section, lines 23-55*<br>The initial introductory sentence has been removed, the study now only focuses on one outcome (level of fall risk), "active" has been removed", and the sentence about ANCOVAs has been revised.

3. References

Alexander, N. B., Ashton-Miller, J. A., Giordani, B., Guire, K., & Schultz, A. B. (2005). Age differences in timed accurate stepping with increasing cognitive and visual demand: A walking trail making test. *The Journals of Gerontology. Series A, Biological Sciences and Medical Sciences*, *60*(12), 1558–1562. https://doi.org/10.1093/gerona/60.12.1558

Bongue, B., Dupré, C., Beauchet, O., Rossat, A., Fantino, B., & Colvez, A. (2011). A screening tool with five risk factors was developed for fall-risk prediction in community-dwelling elderly. *Journal of Clinical Epidemiology*, *64*(10), 1152–1160. https://doi.org/10.1016/j.jclinepi.2010.12.014

Herold, F., Hamacher, D., Schega, L., & Müller, N. G. (2018). Thinking while moving or moving while thinking – Concepts of motor-cognitive training for cognitive performance enhancement. *Frontiers in Aging Neuroscience*, *10*, 228. https://doi.org/10.3389/fnagi.2018.00228

Perrochon, A., & Kemoun, G. (2014). The Walking Trail-Making Test is an early detection tool for mild cognitive impairment. *Clinical Interventions in Aging*, 111. https://doi.org/10.2147/CIA.S53645

Persad, C. C., Jones, J. L., Ashton-miller, J. a, Alexander, N. B., & Giordani, B. (2008). Executive function and gait in older adults with cognitive impairment. *The Journals of Gerontology Series A: Biological Sciences and Medical Sciences*, *63*(12), 1350–1355. https://doi.org/10.1093/gerona/63.12.1350

Schott, N. (2015). Trail Walking Test zur Erfassung der motorisch-kognitiven Interferenz bei älteren Erwachsenen: Entwicklung und Überprüfung der psychometrischen Eigenschaften des Verfahrens. *Zeitschrift Fur Gerontologie Und Geriatrie*, *48*(8), 722–733. https://doi.org/10.1007/s00391-015-0866-3

Van Calster, B., Van Belle, V., Vergouwe, Y., & Steyerberg, E. W. (2012). Discrimination ability of prediction models for ordinal outcomes: Relationships between existing measures and a new measure. *Biometrical Journal. Biometrische Zeitschrift*, *54*(5), 674–685. https://doi.org/10.1002/bimj.201200026

Yamada, M., & Ichihashi, N. (2010). Predicting the probability of falls in community-dwelling elderly individuals using the trail-walking test. *Environmental Health and Preventive Medicine*, *15*(6), 386–391. https://doi.org/10.1007/s12199-010-0154-1
