# Supplementary material for: The Walking Trail Making Test is more accurate than a Dual-Task Walking Test for screening the level of fall risk among community-dwelling older people
Source: PLoS One. 2026 May 21;21(5):e0348875. doi: 10.1371/journal.pone.0348875 (PMC13193416; doi:10.1371/journal.pone.0348875)
Supplement: Attachment — Submitted filename: Review_PONE-D-25_65198_Falls_Walking_TMT.docx [file pone.0348875.s003.docx]

Dear Colleagues,

Thank you for allowing me to review this manuscript concerning how preventing falls is a major issue for older people as aging leads to a decline in executive functions and functional capacities, with an increased fall risk. Here, the authors used a novel dual walking and cognitive task to detect low, moderate, and high risks for falls in a relatively healthy aged population. This manuscript is one of the best I have ever reviewed with a clear and decisive rational and rigorous statistics that were very well thought out and explained in precise detail. I only had a few minor points for the authors to consider to make the manuscript easier to read; however, the manuscript can be accepted as is. I didn’t see some missing information requested by the editorial office which can easily be obtained (see below).

+++++++++++++++++++++++++++++++++++++++++++++++++++++++++++++++++++++++++++++++

*MISSING INFO - Enter a statement with the following details:*

*Initials of the authors who received each award; URL of each funder website; and a statement whether the sponsors or funders played any role in the study design, data collection and analysis, decision to publish, or preparation of the manuscript*

+++++++++++++++++++++++++++++++++++++++++++++++++++++++++++++++++++++++++++++++

Line 70 – The meaning of the sentence as it is written infers that falls occur when a person walks among older people…so anyone walking among a group of older people will most likely fall? I don’t think this is what the authors meant to say that…please consider revising or deleting completely the first 2 sentences in this paragraph…

Lines 75-83 – really important and interesting information about divided attention span and walking speed. Please consider leading with this paragraph and then follow-up with why you need a more accurate tool.

Line 80 – please consider making a new paragraph with the sentence, “Performance in addition DT’s may depend on…

Line 110 – Please consider making this a new paragraph discussing the W-TMT separate from the Trail Walking Test and emphasize the differences between both TMT walking tests.

Line 143-152 – If possible, please provide medication, visual acuity, previous bone fractures, exercise activity, etc. in the inclusion / exclusion criteria…as these can influence the results

Lines 180 – 193 – Very good evaluation and evidence provided to determine fall risk

Lines 196 – 227 – Excellent text characterization of the outcome measures and added distinction of text provided visually in Fig 1…In this way, readers can see the distractor placement

Lines 230 – 233 – Cognitive cost calculations are especially helpful

Lines 236 – 249 – Strong data analyses indicating scientific rigor all things considered – except covariables of medication – if applicable – otherwise, diligence is obvious.

Lines 252- 290 – Very good definitions provided how data was parsed to ensure rigor and accurate methodology were employed. Using ROC/AUC and Youden’s index was especially appreciated given the secondary data acquisition limitation.

Lines – 298-299 – May be useful to include how your data aligns with the standardized norms of the TMT-A and TMT-B *[Tom N Tombaugh, Trail Making Test A and B: Normative data stratified by age and education, Archives of Clinical Neuropsychology, Volume 19, Issue 2, 2004, Pages 203-214, ISSN 0887-6177,* [*https://doi.org/10.1016/S0887-6177(03)00039-8*](https://doi.org/10.1016/S0887-6177(03)00039-8)*]*

Tables and Figures - Visually accurate and easy to interpret

Lines 357 – 364 – Very valuable estimation of the cutoff value which provides a good barometer of an relatively healthy aged population to compare with future populations of patients with movement disorders

Discussion – I would emphasize that the results focused on relatively healthy elders from which standards can be applied against patients with movement disorders in future studies.

Line 395 / 397 – The authors might consider starting a new paragraph for easier readability, “This difference between sensitivity and specificity…” or a new paragraph with… “In the present study, the prevalence of high level of fall risk…”

Line 404 – very good suggestion of primary preventive intervention – will that require a policy change or a grassroots push on Primary care physicians to change the way they intervene with all of their elder patients?

Line 457 – The authors might consider dividing the long paragraph for readers to digest the important discussion points by starting a new paragraph at…”The review conducted by Yogev-Seligmann et al…”

Discussion Overall – If possible, I would like to know the author’s brief view on why women might have had higher fall rates than men – and if there were any correlations between the MOCA or TMT-A-B with motor outcomes with regards to women vs. men? This is not needed for acceptance, however, as the manuscript is valuable as written.
