# Supplementary material for: The Walking Trail Making Test is more accurate than a Dual-Task Walking Test for screening the level of fall risk among community-dwelling older people
Source: PLoS One. 2026 May 21;21(5):e0348875. doi: 10.1371/journal.pone.0348875 (PMC13193416; doi:10.1371/journal.pone.0348875)
Supplement: Attachment — Submitted filename: 02 - Response_to_Reviewers.docx [file pone.0348875.s004.docx]

Dear Pr. Salvatore,

Please find below a point-by-point response to both the editor and reviewers’ comments. We are very grateful to you and the reviewer for the time and effort to review the paper, as well as for very helpful comments. We carefully considered the recommendations and incorporated all relevant suggestions, and we acknowledge the changes significantly improved the paper. The changes are highlighted with **blue ink** in both the revised manuscript and this letter.

**Reviewers’ comments**

Reviewer #1:

The manuscript reflects a very interesting and important topic to investigate to promote primary care physicians to include testing for fall risks in their senior patients. This study was thoughtfully designed with scientific rigor using appropriate as well as accurate data analyses and interpretations. Only very minor edits to make perhaps during the editorial process.

Thank you for allowing me to review this manuscript concerning how preventing falls is a major issue for older people as aging leads to a decline in executive functions and functional capacities, with an increased fall risk. Here, the authors used a novel dual walking and cognitive task to detect low, moderate, and high risks for falls in a relatively healthy aged population. This manuscript is one of the best I have ever reviewed with a clear and decisive rational and rigorous statistics that were very well thought out and explained in precise detail. I only had a few minor points for the authors to consider to make the manuscript easier to read; however, the manuscript can be accepted as is. I didn’t see some missing information requested by the editorial office which can easily be obtained (see below).

We would like to express our gratitude to the reviewer, who provided insightful and constructive comments relative to our initial submission. We carefully considered your suggestions, and you will find point-by-point responses for each of your comments below.

+++++++++++++++++++++++++++++++++++++++++++++++++++++++++++++++++++++++++++++

*MISSING INFO - Enter a statement with the following details:*

*Initials of the authors who received each award; URL of each funder website; and a statement whether the sponsors or funders played any role in the study design, data collection and analysis, decision to publish, or preparation of the manuscript*

This research was funded by the Ligue Auvergne Rhône Alpes de Rugby à XIII and the National Association of Research and Technology by the grant obtained under CIFRE agreement no. 2021/1144. The website addresses for each of the funding organizations are [**http://liguera13.com/**](http://liguera13.com/) and [**https://www.anrt.asso.fr/**](https://www.anrt.asso.fr/) respectively. The Ligue Auvergne Rhône Alpes de Rugby à XIII provided financial support in the form of salaries for the author [R.M.] and research materials. In the study, this funder also organized the recruitment of participants by establishing partnerships with municipal authorities, which then took charge of reaching out to older adults living in their communities. It did not have any additional role in the study design, analysis, decision to publish, or preparation of the manuscript.

+++++++++++++++++++++++++++++++++++++++++++++++++++++++++++++++++++++++++++++

Line 70 – The meaning of the sentence as it is written infers that falls occur when a person walks among older people…so anyone walking among a group of older people will most likely fall? I don’t think this is what the authors meant to say that…please consider revising or deleting completely the first 2 sentences in this paragraph…

Thank you for bringing this to our attention. As you suggested, the first 2 sentences have been rewritten as follows:

“Older adults fall mostly while walking. Consequently, many fall risk assessment tools focus on locomotion performance, particularly in continuous, unperturbed straight-line walking [8,9].” (lines 70-71 in the revised manuscript)

Lines 75-83 – really important and interesting information about divided attention span and walking speed. Please consider leading with this paragraph and then follow-up with why you need a more accurate tool.

Line 80 – please consider making a new paragraph with the sentence, “Performance in addition DT’s may depend on…

As suggested by the reviewer, the second paragraph of the introduction (lines 69–94 in the initial manuscript) has been restructured to address the two comments mentioned above. See below:

“Older adults fall mostly while walking. Consequently, many fall risk assessment tools focus on locomotion performance, particularly in continuous, unperturbed straight-line walking [8,9]. A prospective study showed that a spontaneous walking speed below the threshold of 0.7 m.s^-1^ is associated with a high risk of falling [10]. However, the applicability of this threshold is limited in community-dwelling older people, because they typically have a higher spontaneous walking speed [11]. Using a complex walking task could make such assessments more relevant [12]. Complex walking involves changing direction, navigating on oriented pathways, or avoiding obstacles, which reflect everyday locomotion better than continuous, unperturbed straight-line walking [12,13]. Additionally, complexity is increased by requirement to divide attention between a walking task and a concurrent task, which particularly seems to increase fall occurrences in older people. People who struggle to maintain a conversation while walking are considered to be at a higher risk of falling than those who do not struggle [14]. Adding a cognitive task during simple walking (i.e. *additional* Dual-Task, DT) generally results in a slowing of walking speed [15]. This reflects the “Dual Task Cost” (DTC) (i.e. the cognitive cost of the concurrent cognitive task), and an increase in the DTC is associated with a high risk of falling [16,17].

Performance in *additional* DTs may depend on the prioritization strategy used by the participants (i.e. priority allocated to the locomotor or the *additional* cognitive task) because two distinct goals are present [18]. This prioritization strategy may result in inter-individual and intra-individual variations in performance that do not reflect the true probability of falling, thus limiting the relevance of using an *additional* DT to assess the level of fall risk. Moreover, studies suggest that the complexity of both the locomotor and cognitive tasks influences how relevant *additional* DTs are for fall risk assessment [12,19]. However, even when using an *additional* DT with a complex walking task, studies have obtained mixed results in predicting fall occurrences [20,21]. This may be due to the *additive* aspect of the DTs used, which still allows the participant to prioritize one task over the other [22]. Evidence also suggests that DT testing insufficiently detect people at a high level of fall risk with a sensitivity lower than 55.00% for different types of *additional* DT [23,24]. Thus, the most accurate type of DT remains to be determined, and alternative screening tools should be considered.”

Line 110 – Please consider making this a new paragraph discussing the W-TMT separate from the Trail Walking Test and emphasize the differences between both TMT walking tests.

As suggested by the reviewer, the third paragraph of the introduction (lines 96–120 in the initial manuscript) has been split into two: the first paragraph discusses the Trail Walking Test task, and the second paragraph introduces the WTMT task. See below:

“*Incorporated* cognitive-motor tasks are a promising alternative to *additional* DT, because the cognitive and motor tasks are interdependent. The single and shared overarching goal excludes any possibility of prioritization strategy [22]. Several *incorporated* cognitive-motor tasks involving complex walking have been developed based on neuropsychological assessments targeting fall risk-related factors [12]. Particularly, the Trail Walking Test, a walking task on a 5x5m square setup, has been designed based on the Trail Making Test (TMT) [25], a neuropsychological test assessing Executive Functions (EF) such as processing speed, visuospatial abilities, and cognitive flexibility [26]. As age-related decline in EF is a significant fall risk factor [27,28], this cognitive-motor task showed promising results in the screening of fall risk. A retrospective study found that the performance in the Trail Walking Test discriminated between fallers and non-fallers (based on history of fall) in community-dwelling older people [29]. A prospective study also found the Trail Walking Test more appropriate to predict falls than conventional mobility assessments (e.g. Timed Up and Go test and simple walking test) with a sensitivity of 66.10% and a specificity of 83.90% [30]. Thus, *incorporated* cognitive-motor tasks, designed based on the TMT test, can be considered particularly promising for predicting falls, as has been demonstrated in the case of the Trail Walking Test.

Another test derived from the TMT, the Walking Trail Making Test (W-TMT), has also been developed [31]. Compared to the Trail Walking Test, the W-TMT is quicker to set up and administer; it focuses on linear forward walking, requiring high precision in placing steps on targets while avoiding distractors, rather than on spatial navigation involving continuous changes in direction. The W-TMT consists of walking on targets following a cognitive rule in three separate conditions of increasing complexity: (i) W-TMT-N, that requires to walk on numeric targets in increasing order; (ii) W-TMT-A, the same as W-TMT-N but with distractors to avoid, and (iii) W-TMT-B, that requires to alternate between numeric and alphabetic targets (increasing and alphabetic order respectively) while avoiding distractors. Performance in the W-TMT is measured using execution time in each condition and Delta-WTMT (i.e. the cognitive cost of increased complexity between the B and A conditions). Interestingly, the execution time in the W-TMT-B condition and the Delta-WTMT were both significantly increased in people with EF-related cognitive impairments, a significant fall risk factor [31–33]. However, no study assessed whether the W-TMT performance could differentiate between different levels of fall risk in community-dwelling older people, compared to an *additional* DT involving simple walking.”

Line 143-152 – If possible, please provide medication, visual acuity, previous bone fractures, exercise activity, etc. in the inclusion / exclusion criteria…as these can influence the results

In this study, medication was not part of the inclusion/exclusion criteria and participants with visual problems had to wear their common correction glasses. However, participants had to undergo a medical examination by their common primary care physician to confirm that there were no contraindications to physical activity.

In the “Participants” section, the following clarification regarding visual acuity has been added:

“If needed, participants were instructed to wear their common correction glasses.” (lines 160-161 in the revised manuscript)

Please also see our response to the comment regarding lines 246-249 (below).

Lines 180 – 193 – Very good evaluation and evidence provided to determine fall risk

Thank you.

The SRC-CES questionnaire is a “Gold standard” developed by the French health-care system. This tool was designed with the purpose of identifying the most relevant and independent fall risk factors to provide a rapid screening tool.

Bongue, B., Dupré, C., Beauchet, O., Rossat, A., Fantino, B., & Colvez, A. (2011). A screening tool with five risk factors was developed for fall-risk prediction in community-dwelling elderly. Journal of clinical epidemiology, 64(10), 1152–1160. <https://doi.org/10.1016/j.jclinepi.2010.12.014>

Lines 196 – 227 – Excellent text characterization of the outcome measures and added distinction of text provided visually in Fig 1…In this way, readers can see the distractor placement

Thank you.

Lines 230 – 233 – Cognitive cost calculations are especially helpful

Thank you.

Lines 236 – 249 – Strong data analyses indicating scientific rigor all things considered – except covariables of medication – if applicable – otherwise, diligence is obvious.

Thank you.

The SRC-CES tool was used to define the different groups used in the analyses of the C-index and the ROC curve. One of its five questions specifically concerns medication use and is written as follows: “Do you take any psychotropic medications (like antidepressants, sleeping pills, antipsychotics, anxiolytics)?”. When an older adult answers YES to this question, 1 point is added to their fall risk score.

In our sample of 101 community-dwelling older adults, only 19 people (18.8%) reported taking these medications. Given how the SRC-CES tool is designed, most of these individuals (17, *i.e.* 89.47%) were at moderate or high risk of falling. More precisely, these individuals are distributed as follows across the three groups:

- Low fall risk: 2 out of 34 people (or 5.88%)
- Moderate fall risk: 13 out of 54 people (or 24.07%)
- High fall risk: 4 out of 13 people (or 30.77%)

The difference observed in the distribution of individuals taking psychotropic medications across the three groups is just barely significant (*χ^2^*=5.92, *p*=.052) but may not be representative considering the very small sample within each group.

Thanks to the fall risk assessment tool used in this study, the 3 groups defined based on this score already consider the use of psychotropic medications. Thus, we decided not to include these additional details in the revised manuscript.

Lines 252 – 290 – Very good definitions provided how data was parsed to ensure rigor and accurate methodology were employed. Using ROC/AUC and Youden’s index was especially appreciated given the secondary data acquisition limitation.

Thank you.

Lines 298 – 299 – May be useful to include how your data aligns with the standardized norms of the TMT-A and TMT-B *[Tom N Tombaugh, Trail Making Test A and B: Normative data stratified by age and education, Archives of Clinical Neuropsychology, Volume 19, Issue 2, 2004, Pages 203-214, ISSN 0887-6177,* [*https://doi.org/10.1016/S0887-6177(03)00039-8*](https://doi.org/10.1016/S0887-6177(03)00039-8)*]*

As noted in the “Methods” section, this study is an ancillary study of the 13EVAL cluster-randomized controlled trial (lines 141–142). In the 13EVAL study, we have compared the performance observed in the cognitive tests to normative standard. Since it is recommended to use norms derived from a population whose characteristics are as close as possible to those of the population being assessed, we used norms obtained from a sample of elderly individuals of French nationality. In the revised version of the manuscript, we have reported the results of a comparison based on such norms, citing the following study by Amieva et al., (2009):

Amieva, H., Le Goff, M., Stoykova, R., Lafont, S., Ritchie, K., Tzourio, C., Fabrigoule, C., & Dartigues, J.-F. (2009). Trail Making Test A et B (version sans correction des erreurs) : Normes en population chez des sujets âgés, issues de l’étude des trois Cités: *Revue de Neuropsychologie Neurosciences Cognitives et Cliniques*, *Volume 1*(3), 210‑220. <https://doi.org/10.1684/nrp.2009.0031>

These norms consider the person’s gender, age, and level of education. This level of education considers both the number of years of schooling and the completion of the French Baccalaureate (Bac). According to these criteria, two categories are distinguished: (1) individuals with fewer than 12 years of schooling and (2) those who have completed the French baccalaureate (Bac) and have at least 12 years of schooling. Finally, these norms are provided for the 5th, 10th, 25th, 50th, and 75th percentiles.

--------------

The TMT standardized norms, developed by Tombaugh (2004), were established using a sample of Canadian citizens adults. Unlike those of Amieva et al. (2009), they do not take the individual’s gender into account. For the level of education, only a threshold corresponding to 12 years of schooling is used to distinguish between two groups: (1) individuals with 12 years of schooling or less and (2) individuals with more than 12 years. In addition, these norms are provided for every 10th percentile.

Thus, to compare the results with those observed using the norms of Amieva et al. (2009), we defined the following four normative categories:

- below the 10th percentile: deficient.

- between the 10th and 20th percentiles, inferior.

- between the 20th and 80th percentiles, average.

- above the 80th percentile, superior.

The results of this classification according to these two normative standards are presented in Tables A and B (for the TMT-A) and Tables C and D (for the TMT-B) on pages 7 and 8 of this document.

**Measured performance in the TMT-A:**

**classification of participants according to the two normative standards.**

Use of the standardized norms by Amieva et al. (2009)

**Table A.** TMT-A: repartition of each normative category corrected by age, gender and educational level, n (%).

|  | Level of fall risk | | |
| --- | --- | --- | --- |
| Normative category  *Percentile, name* | **High**  **(N=13)** | **Moderate**  **(N=54)** | **Low**  **(N=34)** |
| <10, deficit | 0(0.00) | 3(5.56) | 1(2.94) |
| [10-25[, inferior | 0(0.00) | 3(5.56) | 1(2.94) |
| [25-75], average | 3(23.08) | 8(14.81) | 9(26.47) |
| >75, superior | 10(76.92) | 40(74.07) | 23(67.65) |

*Note.* Regarding Amieva et al., 2009 classification, participants are considered in deficit from the 10^th^ percentile.

Use of the standardized norms by Tombaugh (2004)

**Table B.** TMT-A: repartition of each normative category corrected by age and educational level, n (%).

|  | Level of fall risk | | |
| --- | --- | --- | --- |
| Normative category  *Percentile, name* | **High**  **(N = 13)** | **Moderate**  **(N = 54)** | **Low**  **(N = 34)** |
| <10, deficit | 0(0.00) | 6(11.11) | 1(2.94) |
| [10-20[, inferior | 0(0.00) | 1(1.85) | 2(5.88) |
| [20-80], average | 6(46.15) | 33(61.11) | 16(47.06) |
| >80, superior | 7(53.85) | 14(25.93) | 15(44.12) |

*Note.* Participants are considered in deficit from the 10^th^ percentile.

Tables A and B show similar distributions of individuals across the four normative classes considered, but differences are observed in the average and superior classes. Using the norms produced by Tombaugh (2004), more individuals score in the average normative class, and fewer in the superior class. These differences could be explained by the differences (reported above) in how these norms were developed (*i.e.*, without considering gender and whether an individual holds the French Baccalaureate when determining the level of education).

Nevertheless, **regardless of the norms used**, the distribution of individuals across the different normative classes **did not differ statistically based on the three levels of fall risk**. According to each of the norms, the results of the chi-square tests are respectively equal to:

- *X^2^*_(6)_=3.58, *p*=.734 (norms of Amieva et al., 2009).
- *X^2^*_(6)_=8.90, *p*=.179 (norms of Tombaugh, 2004).

**Measured performance in the TMT-B:**

**classification of participants according to the two normative standards.**

Use of the standardized norms by Amieva et al. (2009)

**Table C.** TMT-B: repartition of each normative category corrected by age, gender and educational level, n (%).

|  | Level of fall risk | | |
| --- | --- | --- | --- |
| Normative category  *Percentile, name* | **High**  **(N = 13)** | **Moderate**  **(N = 54)** | **Low**  **(N = 34)** |
| <10, deficit | 1(7.69) | 4(7.41) | 0(0.00) |
| [10-25[, inferior | 0(0.00) | 3(5.56) | 3(8.82) |
| [25-75], average | 7(53.84) | 17(31.48) | 10(29.41) |
| >75, superior | 5(38.46) | 30(55.56) | 21(61.76) |

*Note.* Regarding Amieva et al., 2009 classification, participants are considered in deficit from the 10^th^ percentile.

Use of the standardized norms by Tombaugh (2004)

**Table D.** TMT-B: repartition of each normative category corrected by age and educational level, n (%).

|  | Level of fall risk | | |
| --- | --- | --- | --- |
| Normative category  *Percentile, name* | **High**  **(N=13)** | **Moderate**  **(N=54)** | **Low**  **(N=34)** |
| <10, deficit | 0(0.00) | 5(9.26) | 1(2.94) |
| [10-20[, inferior | 2(0.00) | 3(5.56) | 0(0.00) |
| [20-80], average | 6(46.15) | 19(35.18) | 13(38.24) |
| >80, superior | 5(38.46) | 27(50.00) | 20(58.82) |

*Note.* Participants are considered in deficit from the 10^th^ percentile.

As with the TMT-A, Tables C and D show similar distributions of individuals across the four normative classes. In each fall risk group, only a few individuals are classified differently: 2, 4, and 3 for the high, moderate, and low fall risk groups, respectively. As explained above, the way the norms were developed could explain these differences.

Nevertheless, **regardless of the norms used**, the distribution of individuals across the different normative classes **did not differ statistically based on the three levels of fall risk**. According to each of the norms, the results of the chi-square tests are respectively equal to:

- *X^2^*_(6)_=6.55, *p*=.364 (norms of Amieva et al., 2009).
- *X^2^*_(6)_=8.90, *p*=.179 (norms of Tombaugh, 2004).

For the revised version of the manuscript, we propose adding additional results (as Supplementary Results) using the norms for the TMT-A and TMT-B provided by Amieva et al. (2009). These norms are highly consistent with our sample of French older adults, in which 84% are women. They were also derived from French seniors. They include gender, and the definition of educational level considers whether the individual has obtained the French Baccalaureate (in addition to the number of years of schooling).

The following changes have been made to different subsections of the METHODS section.

**“Cognitive flexibility” section** (lines 187-193 in the revised manuscript)

“Execution times were compared to the normative standard of the TMT-A and TMT-B recorded in 8,995 French older adults living in the community by Amieva et al., (2009) [37]. These norms consider the person’s gender, age, and level of education. Both the number of years of schooling and the attainment of the French Baccalaureate (Bac) were used to define the level of education. Using the provided percentiles, four normative classes were defined: deficient (below the 10th), inferior (between the 10th and 25th), average (from the 25th to the 75th), and superior (above the 75th).”

**“Data analysis” section** (lines 258-260 in the revised manuscript)

“Chi-square tests were used to compare the distributions of different categorical variables across the three levels of fall risk: gender, number of previous falls, and normative performance classes on the TMT.”

**Supplementary Results**

A reference to the supplementary results has been added to the revised manuscript on lines 528–529 as follows:

**Supporting information**

**S1 Results.** **Normative classes for the Trail Making Test.**

They are presented in the Word document titled “04 - S1 Normative classes for the Trail Making Test,” see below.

In the total sample, regarding TMT-A, 4 of the participants were classified as having a “deficit”: 1 (or 2.94%) in the low level of fall risk and 3 (or 5.56%) in the moderate level of fall risk (see Table 4). Regarding TMT-B, only 5 participants were classified as having a “deficit”: 4 (or 7.41%) in the moderate level of fall risk and 1 (or 7.69%) in the high level of fall risk (see Table 5). The overall distribution for each normative class did not show any statistical difference across the three levels of fall risk (for TMT-A, *X^2^*_(6)_ =3.58, *p*=.734 and for TMT-B, *X^2^*_(6)_ =6.55, *p*=.364).

Table 4. TMT-A: repartition of each normative category corrected by age, gender and educational level, n (%).

|  | Level of fall risk | | |
| --- | --- | --- | --- |
| Normative category  *Percentile, name* | **High**  (N=13) | **Moderate**  (N=54) | **Low**  (N=34) |
| <10, deficit | 0(0.00) | 3(5.56) | 1(2.94) |
| [10-25[, inferior | 0(0.00) | 3(5.56) | 1(2.94) |
| [25-75], average | 3(23.08) | 8(14.81) | 9(26.47) |
| >75, superior | 10(76.92) | 40(74.07) | 23(67.65) |

*Note.* In accordance with the normative standard proposed by Amieva et al. (2009), participants are considered in deficit from the 10^th^ percentile.

**Table 5.** TMT-B: repartition of each normative category corrected by age, gender and educational level, n (%).

|  | Level of fall risk | | |
| --- | --- | --- | --- |
| Normative category  *Percentile, name* | **High**  **(N = 13)** | **Moderate**  **(N = 54)** | **Low**  **(N = 34)** |
| <10, deficit | 1(7.69) | 4(7.41) | 0(0.00) |
| [10-25[, inferior | 0(0.00) | 3(5.56) | 3(8.82) |
| [25-75], average | 7(53.84) | 17(31.48) | 10(29.41) |
| >75, superior | 5(38.46) | 30(55.56) | 21(61.76) |

*Note.* In accordance with the normative standard proposed by Amieva et al. (2009), participants are considered in deficit from the 10^th^ percentile.

Tables and Figures – Visually accurate and easy to interpret

Thank you.

Lines 357 – 364 – Very valuable estimation of the cutoff value which provides a good barometer of a relatively healthy aged population to compare with future populations of patients with movement disorders

Thank you.

Discussion – I would emphasize that the results focused on relatively healthy elders from which standards can be applied against patients with movement disorders in future studies.

Thank you for your comment. The first paragraph of the Discussion has been revised accordingly (lines 398-400 in the revised manuscript).

“This result suggests that *incorporated* cognitive-motor tasks are more accurate than *additional* cognitive-motor tasks for detecting people at a high level of fall risk among relatively healthy community-dwelling older adults.”

Line 395 / 397 – The authors might consider starting a new paragraph for easier readability, “This difference between sensitivity and specificity…” or a new paragraph with… “In the present study, the prevalence of high level of fall risk…”

Thank you.

Following your suggestion we have split the paragraph “*The W-TMT-B, a tool to detect...*” at line 416. The second paragraph created begins with “*In the present study, the prevalence of high level of fall risk....*”.

Line 404 – very good suggestion of primary preventive intervention – will that require a policy change or a grassroots push on Primary care physicians to change the way they intervene with all of their elder patients?

Thank you for your comment and question.

Since 2016, French legislation allows general practitioners to prescribe physical activity programs for individuals with chronic diseases and older adults with loss of autonomy. Although this law supports the use of physical activity in primary and secondary prevention, prescribing practices for this non-pharmacological therapy remain limited and require further development. To increase its adoption, targeted strategies could include training physicians on evidence-based physical activity guidelines, fostering interdisciplinary collaborations with physical activity specialists (e.g., physiotherapists, sports educators), and raising public awareness about the benefits of physical activity for chronic disease management. We hope that, with this study, we can share scientific evidence that will encourage these future changes.

Line 457 – The authors might consider dividing the long paragraph for readers to digest the important discussion points by starting a new paragraph at… “The review conducted by Yogev-Seligmann et al…”

Thank you.

Following your suggestion we have split this paragraph at line 479. This new paragraph expands on the second idea, explaining how the task might have been prioritized. It begins with the sentence “*Another possibility is that participants may have prioritized...*”.

Discussion Overall – If possible, I would like to know the author’s brief view on why women might have had higher fall rates than men – and if there were any correlations between the MOCA or TMT-A-B with motor outcomes with regards to women vs. men? This is not needed for acceptance, however, as the manuscript is valuable as written.

Thank you for this insightful comment. Below are some arguments that come from studies that we read in the conceptualization of this project that might answer your interrogation.

Higher fall rates among older women reflect the interplay of physiological, medical, and behavioral risk factors (Stevens & Sogolow, 2005). Physiologically, age-related sarcopenia affects women earlier due to lower baseline muscle mass, accelerating balance impairment (Moreland et al., 2004). Demographically, women’s greater longevity results in overrepresentation in the highest-risk age groups (≥80 years). Medically, women experience higher rates of mobility-limiting chronic conditions (e.g., osteoarthritis, osteoporosis) and polypharmacy, particularly psychotropic medications, which increase fall risk through side effects like dizziness (Leipzig et al., 1999). Behaviorally, domestic routines—often involve multitasking, bending, and navigating hazardous surfaces—further elevate exposure to environmental fall risks (Stevens & Sogolow, 2005).

Leipzig, R. M., Cumming, R. G., & Tinetti, M. E. (1999). Drugs and falls in older people: A systematic review and meta-analysis: I. Psychotropic drugs. *Journal of the American Geriatrics Society*, *47*(1), 30–39. https://doi.org/10.1111/j.1532-5415.1999.tb01898.x

Moreland, J. D., Richardson, J. A., Goldsmith, C. H., & Clase, C. M. (2004). Muscle weakness and falls in older adults: A systematic review and meta-analysis. *Journal of the American Geriatrics Society*, *52*(7), 1121–1129. https://doi.org/10.1111/j.1532-5415.2004.52310.x

Stevens, J. A., & Sogolow, E. D. (2005). Gender differences for non-fatal unintentional fall related injuries among older adults. *Injury Prevention*, *11*(2), 115–119. https://doi.org/10.1136/ip.2004.005835

--------------

In our sample, the correlation between spontaneous walking speed and cognitive outcomes is significant only for women but remains low (see Table below). This is probably because they appear to be relatively healthy. Given the small correlation results, we decided not to include those in the revised manuscript.

|  | Women  (N=85) | Men  (N=16) |
| --- | --- | --- |
| MoCA / Gait speed | R^2^=.19, *p*=.08 | R^2^=.33, *p*=.22 |
| TMT-A / Gait speed | R^2^=-.29, *p*=.007 | R^2^=.11, *p*=.68 |
| TMT-B / Gait speed | R^2^=-.24, *p*=.028 | R^2^=.06, *p*=.83 |

**Reviewer’s Responses to Questions**

**1.** Is the manuscript technically sound, and do the data support the conclusions?

The manuscript must describe a technically sound piece of scientific research with data that supports the conclusions. Experiments must have been conducted rigorously, with appropriate controls, replication, and sample sizes. The conclusions must be drawn appropriately based on the data presented.

Reviewer #1: Yes

Thank you.

**2.** Has the statistical analysis been performed appropriately and rigorously?

Reviewer #1: Yes

Thank you.

**3.** Have the authors made all data underlying the findings in their manuscript fully available?

The [PLOS Data policy](http://track.editorialmanager.com/CL0/http:%2F%2Fwww.plosone.org%2Fstatic%2Fpolicies.action%23sharing/1/010f019d4d3e94ea-b4701b1b-3966-4e3f-a8b6-8a545dcfecd8-000000/RLyYNhs0DJ-n2efJDjOn-G1JIerkwLd758khhczDjaE=257) requires authors to make all data underlying the findings described in their manuscript fully available without restriction, with rare exception (please refer to the Data Availability Statement in the manuscript PDF file). The data should be provided as part of the manuscript or its supporting information, or deposited to a public repository. For example, in addition to summary statistics, the data points behind means, medians and variance measures should be available. If there are restrictions on publicly sharing data —e.g. participant privacy or use of data from a third party— those must be specified.

Reviewer #1: Yes

The selected public repository is [www.zenodo.org](http://www.zenodo.org)

MAUTI, R., FARGIER, P., & CHABAUD, P. (2026). 13EVAL_Older_Adults_FALLS [Data set]. Zenodo. <https://doi.org/10.5281/zenodo.19665098>

**4.** Is the manuscript presented in an intelligible fashion and written in standard English?

PLOS ONE does not copyedit accepted manuscripts, so the language in submitted articles must be clear, correct, and unambiguous. Any typographical or grammatical errors should be corrected at revision, so please note any specific errors here.

Reviewer #1: Yes

Thank you.

**Other revisions**

**1.** The affiliations of the five authors affiliated with the Université Lyon 1 have been updated in accordance with our university’s latest guidelines.

The authors in question are as follows: Rafael Mauti^1^, MSc; Melanie Gallot^1^, Ph.D.; Thomas Gilbert, M.D.-Ph.D.^6,7^; Patrick Fargier^1¶^, Ph.D.; Pascal Chabaud^1¶^, Ph.D.

Their affiliations, as revised in accordance with these guidelines, are now:

^1^Université Lyon 1, Université Jean Monnet, Univ. Savoie Mont Blanc, LIBM, EA 7424, Villeurbanne, France

^6^Université Lyon 1, INSERM, RESHAPE, UMR S1290, Lyon, France

^7^Université Lyon 1, Hospices Civils de Lyon, Hop Lyon Sud, Pierre-Bénite, France

**2.** Adjustment of MoCA scores to account for educational level

In the initial version of the manuscript, raw MoCA scores were presented. With the addition of supplementary data regarding normative classes for TMT performance, we now present MoCA scores adjusted for educational level, in accordance with the recommendations of Nasreddine et al. (2005). This involves adding 1 point to the raw score when the number of years of schooling is 12 or fewer. Although the observed averages are slightly higher, all results remain unchanged.

| **Participants**  **(N)** | **Raw MoCA score**  **(mean ± SD)** | **Adjusted MoCA score**  **(mean ± SD)** |
| --- | --- | --- |
| Total sample  (N=101) | 24.80±3.02 | 25.07±3.10 |
| Low level of fall risk  (N=34) | 24.82±2.71 | 25.09±2.84 |
| Moderate level of fall risk  (N=54) | 24.74±3.19 | 25.02±3.24 |
| High level of fall risk  (N=13) | 25.00±3.27 | 25.23±3.42 |

The following changes have been made to various sections of the manuscript.

**Methods section, lines 178-179**

“MoCA scores were adjusted for educational level according to the recommendations of Nasreddine et al. (2005) [36].”

**Results section, lines 312-314**

“The average MoCA score was 25.07±3.10; *range* [18-30], with 50.50% (N=51) of all participants with a MoCA score below the threshold of 26/30 suggesting a probable cognitive impairment.”

**Modification of the row for the MoCA in Table 1**

**Table 1.** **Baseline differences in continuous variables among the three levels of fall risk given by the SRC-CES.**

|  | Level of fall risk | | |  |  |
| --- | --- | --- | --- | --- | --- |
| Variable | **High**  **(N = 13)** | **Moderate**  **(N = 54)** | **Low**  **(N = 34)** | ***F*_2,98_** | ***p*** |
| Age (years) | 78.06 (±5.38) | 75.89 (±6.31) | 75.01 (±6.35) | 1.13 | .32 |
| Weight (kg) | 64.96 (±7.74) | 64.47 (±11.59) | 68.03 (±11.38) | 1.10 | .34 |
| BMI (kg.m^-2^) | 25.85 (±3.34) | 24.92 (±4.04) | 23.95 (±3.43) | 1.38 | .26 |
| Education (years) | 10.15 (±4.67) | 10.80 (±3.85) | 12.12 (±3.58) | 1.71 | .19 |
| Spontaneous Walking Speed (m.s^-1^) | 1.09 (±0.12) | 1.12 (±0.19) | 1.18 (±0.21) | 1.63 | .20 |
| MoCA (score /30)^●^ | 25.23 (±3.42) | 25.02 (±3.24) | 25.09 (±2.84) | 0.02 | .98 |
| TMT-A (s) | 37.37 (±11.32) | 41.91 (±17.43) | 38.30 (±12.07) | 0.84 | .43 |
| TMT-B (s) | 111.01 (±68.80) | 96.13 (±49.38) | 79.53 (±32.32) | 2.42 | .09 |
| Delta-TMT (B-A, s) | 73.64 (±60.59)^†^ | 54.21 (±40.35) | 41.23 (±26.32) | 3.26 | .043 |

Mean (±SD); ^●^MoCA scores adjusted for educational level; ^†^Significant difference between high and low level in *post-hoc* comparisons

**PLOS One – Journal Requirements**

When submitting your revision, we need you to address these additional requirements.

**1.** Please ensure that your manuscript meets PLOS ONE's style requirements, including those for file naming. The PLOS ONE style templates can be found at

<https://journals.plos.org/plosone/s/file?id=wjVg/PLOSOne_formatting_sample_main_body.pdf>

<https://journals.plos.org/plosone/s/file?id=ba62/PLOSOne_formatting_sample_title_authors_affiliations.pdf>

The manuscript has been formatted in accordance with PLOS ONE’s style requirements.

**2.** Thank you for stating the following financial disclosure:

“*This research was funded by the Ligue Auvergne Rhône Alpes de Rugby à XIII and the National Association of Research and Technology by the grant obtained under CIFRE agreement no. 2021/1144.*”

**Please state what role the funders took in the study**. If the funders had no role, please state: "*The funders had no role in study design, data collection and analysis, decision to publish, or preparation of the manuscript.*"

If this statement is not correct you must amend it as needed. **Please include this amended Role of Funder statement in your cover letter**; we will change the online submission form on your behalf.

**3.** Thank you for stating the following financial disclosure:

“*This research was funded by the Ligue Auvergne Rhône Alpes de Rugby à XIII and the National Association of Research and Technology by the grant obtained under CIFRE agreement no. 2021/1144.*”

We note that one or more of the authors is affiliated with the funding organization, indicating the funder may have had some role in the design, data collection, analysis or preparation of your manuscript for publication; in other words, the funder played an indirect role through the participation of the co-authors. If the funding organization did not play a role in the study design, data collection and analysis, decision to publish, or preparation of the manuscript and only provided financial support in the form of authors' salaries and/or research materials, please do the following:

a. **Review your statements relating to the author’s contributions** and ensure you have specifically and accurately indicated the role(s) that these authors had in your study. **These amendments should be made in the online form**.

b. **Confirm in your cover letter** that you agree with the following statement, and we will change the online submission form on your behalf:

“*The funder provided support in the form of salaries for authors [insert relevant initials] but did not have any additional role in the study design, data collection and analysis, decision to publish, or preparation of the manuscript. The specific roles of these authors are articulated in the ‘author contributions’ section.*”

Considering the comments regarding the financial disclosure (items 2 and 3), the following amended statement was added to the cover letter:

“This research was funded by the Ligue Auvergne Rhône Alpes de Rugby à XIII and the National Association of Research and Technology by the grant obtained under CIFRE agreement no. 2021/1144. The website addresses for each of the funding organizations are [**http://liguera13.com/**](http://liguera13.com/) and [**https://www.anrt.asso.fr/**](https://www.anrt.asso.fr/) respectively. The Ligue Auvergne Rhône Alpes de Rugby à XIII provided financial support in the form of salaries for the author [R.M.] and research materials. In the study, this funder also organized the recruitment of participants by establishing partnerships with municipal authorities, which then took charge of reaching out to older adults living in their communities. It did not have any additional role in the study design, analysis, decision to publish, or preparation of the manuscript. The specific roles of these authors [A.P.] are articulated in the ‘author contributions’ section.”

**4.** When completing the data availability statement of the submission form, you indicated that you will make your data available on acceptance. We strongly recommend all authors decide on a data sharing plan before acceptance, as the process can be lengthy and hold up publication timelines. Please note that, though access restrictions are acceptable now, your entire data will need to be made freely accessible if your manuscript is accepted for publication. This policy applies to all data except where public deposition would breach compliance with the protocol approved by your research ethics board. If you are unable to adhere to our open data policy, please kindly revise your statement to explain your reasoning and we will seek the editor's input on an exemption. Please be assured that, once you have provided your new statement, the assessment of your exemption will not hold up the peer review process.

As indicated in the submission, all data underlying the findings in the manuscript will be available on a public repository. We have chosen the following repository: [www.zenodo.org](http://www.zenodo.org)

MAUTI, R., FARGIER, P., & CHABAUD, P. (2026). 13EVAL_Older_Adults_FALLS [Data set]. Zenodo. <https://doi.org/10.5281/zenodo.19665098>

**5.** If the reviewer comments include a recommendation to cite specific previously published works, please review and evaluate these publications to determine whether they are relevant and should be cited. There is no requirement to cite these works unless the editor has indicated otherwise.

**6.** Please review your reference list to ensure that it is complete and correct. If you have cited papers that have been retracted, please include the rationale for doing so in the manuscript text, or remove these references and replace them with relevant current references. Any changes to the reference list should be mentioned in the rebuttal letter that accompanies your revised manuscript. If you need to cite a retracted article, indicate the article’s retracted status in the References list and also include a citation and full reference for the retraction notice.

The reference list has been updated with the references specified in this rebuttal letter. It has also been verified.

**7.** To ensure your figures meet our technical requirements, please review our figure guidelines: <https://journals.plos.org/plosone/s/figures>

You may also use PLOS’s free figure tool, NAAS, to help you prepare publication quality figures: <https://journals.plos.org/plosone/s/figures#loc-tools-for-figure-preparation>

NAAS will assess whether your figures meet our technical requirements by comparing each figure against our figure specifications.

All figures in the article meet the technical requirements.
